# Cross-evaluation of wearable data for use in Parkinson’s disease research: a free-living observational study on Empatica E4, Fitbit Sense, and Oura

**DOI:** 10.1186/s12938-025-01353-0

**Published:** 2025-02-21

**Authors:** Haakon Reithe, Brice Marty, Juan C. Torrado, Elise Førsund, Bettina S. Husebo, Ane Erdal, Simon U. Kverneng, Erika Sheard, Charalampos Tzoulis, Monica Patrascu

**Affiliations:** 1 Centre for Elderly and Nursing Home Medicine, Department of Global Public Health and Primary Care, University of Bergen, Bergen, Norway; 2 Neuro-SysMed Center, Department of Global Public Health and Primary Care, University of Bergen, Bergen, Norway; 3 Norwegian Computing Centre, Oslo, Norway; 4 The Hospital Pharmacy in Bergen, Haukeland University Hospital, Bergen, Norway; 5 Neuro-SysMed Center, Department of Neurology, Haukeland University Hospital, Bergen, Norway; 6 Department of Clinical Medicine, University of Bergen, Bergen, Norway; 7 K.G Jebsen Center for Translational Research in Parkinson’s Disease, University of Bergen, Bergen, Norway; 8 Complex Systems Laboratory, Department of Automatic Control and Systems Engineering, University Politehnica of Bucharest, Bucharest, Romania

**Keywords:** Wearable devices, Parkinson’s disease, Cross-evaluation, System usability, Smart wearables, Multi-modal sensing

## Abstract

**Background:**

Established assessment scales used for Parkinson’s disease (PD) have several limitations in tracking symptom progression and fluctuation. Both research and commercial-grade wearables show potential in improving these assessments. However, it is not known whether pervasive and affordable devices can deliver reliable data, suitable for designing open-source unobtrusive around-the-clock assessments. Our aim is to investigate the usefulness of the research-grade wristband Empatica E4, commercial-grade smartwatch Fitbit Sense, and the Oura ring, for PD research.

**Method:**

The study included participants with PD (*N* = 15) and neurologically healthy controls (*N* = 16). Data were collected using established assessment scales (Movement Disorders Society Unified Parkinson’s Disease Rating Scale, Montreal Cognitive Assessment, REM Sleep Behavior Disorder Screening Questionnaire, Hoehn and Yahr Stage), self-reported diary (activities, symptoms, sleep, medication times), and 2-week digital data from the three devices collected simultaneously. The analyses comprised three steps: preparation (device characteristics assessment, data extraction and preprocessing), processing (data structuring and visualization, cross-correlation analysis, diary comparison, uptime calculation), and evaluation (usability, availability, statistical analyses).

**Results:**

We found large variation in data characteristics and unsatisfactory cross-correlation. Due to output incongruences, only heart rate and movement could be assessed across devices. Empatica E4 and Fitbit Sense outperformed Oura in reflecting self-reported activities. Results show a weak output correlation and significant differences. The uptime was good, but Oura did not record heart rate and movement concomitantly. We also found variation in terms of access to raw data, sampling rate and level of device-native processing, ease of use, retrieval of data, and design. We graded the system usability of Fitbit Sense as good, Empatica E4 as poor, with Oura in the middle.

**Conclusions:**

In this study we identified a set of characteristics necessary for PD research: ease of handling, cleaning, data retrieval, access to raw data, score calculation transparency, long battery life, sufficient storage, higher sampling frequencies, software and hardware reliability, transparency. The three analyzed devices are not interchangeable and, based on data features, none were deemed optimal for PD research, but they all have the potential to provide suitable specifications in future iterations.

**Supplementary Information:**

The online version contains supplementary material available at 10.1186/s12938-025-01353-0.

## Background

Parkinson’s disease (PD) is the most common neurodegenerative movement disorder [1, 2]. Clinically, PD is characterized by progressive motor dysfunction, including bradykinesia, tremor, and rigidity, and a multitude of non-motor symptoms, including hyposmia, autonomic dysregulation, neuropsychiatric disorders, gastrointestinal dysmotility, cognitive impairment and dementia [3, 4]. In addition, a large proportion of individuals with PD exhibit sleep disorders, predominantly rapid eye movement (REM) sleep behavior disorder (RBD), characterized by loss of REM-sleep atonia and dream enactment [5]. Both motor and non-motor symptoms of PD show highly interindividual variability in terms of composition, severity, and progression rates [6]. Moreover, many of these symptoms exhibit diurnal fluctuations, which may or may not be associated with dopaminergic treatment [7–9].

Established clinical scales, such as the Movement Disorders Unified Parkinson’s Disease Rating Scale (MDS-UPDRS) are routinely used to assess disease severity in PD. However, these scales suffer of important limitations in tracking symptom progression and fluctuation [10–12]. First, as they require neurological assessment, they only provide a limited number (typically 1–3 per year) of static snapshots of the individual state. This is particularly problematic for motor symptoms which vary significantly depending on the dose and time from last intake of dopaminergic treatment. Symptom diaries can complement the scales, but they have limited accuracy and reliability due to variation in adherence and reporting time [13, 14]. Second, these scales are prone to substantial inter- and intra-rater variability [15], limiting their reliability in long-term disease monitoring. Thus, there is currently a need for developing objective means of tracking PD symptoms in free-living conditions.

Wearable sensors have shown potential in improving the assessment of PD symptoms by providing high-resolution quantitative data on prevalence, severity, and treatment response over time [10, 11, 16–18]. Devices such as the Motor fluctuations Monitor for Parkinson’s Disease (MM4PD) or the Parkinson's KinetiGraph (PKG) have shown good correlation with clinical scales [19], potential to measure bradykinesia and dyskinesia [20], tremors [21], fluctuations [22] and immobility [23] under real-world conditions.

From the perspective of the person with PD, user friendliness, affordability, small size and lightweight style, durability, energy efficiency, and waterproof properties are important in a wearable. From the perspective of the PD researcher, high sensitivity (high sampling rate), costs and reproducibility are also critical [24–27]. For instance, while PKG has shown good results, the manufacturer has received criticism for not disclosing the algorithm that calculates their scores, making it unavailable for others to validate [28]. In a recent review [29], devices such as the PDMonitor, STAT-ON, Kinesia360 and Feet Me have been identified, along with PKG, as among the most used in Europe for monitoring and treatment management in PD. These wearables are either wrist/ankle-worn, clipped on the belt, or inserted in shoes, and have shown moderate-to-high correlation with clinical assessment scales or symptom diaries. Out of seven total wearable devices evaluated by [29], four are specific to PD symptoms, while the other three are specific to gait, posture and walking parameters. For clinical studies that aim to investigate other physiological parameters, additional sensors would be necessary, which has raised the question on whether general-purpose devices could be more easily adapted across clinical trials by allowing the computation of a wider range of outcomes.

All these factors make consumer-grade devices very attractive for long-term use, whereas research-grade devices have been described as designed for short-term use [30]. However, it is unknown whether these pervasive and affordable general-purpose devices can deliver reliable data and could this data be used in designing open-source unobtrusive around-the-clock assessments [19, 31–33]. For instance, previous research has shown large variation in data storage, access to data and software development kits (SDK) between Google Fit + Android Wear, Apple Health and S-Health, three large software platforms for consumer wearables [34]. This paints a picture of a large and complex world of wearables sensing devices.

Therefore, the aim of the current study is twofold: (1) investigate the utility of three general-purpose wearable devices for PD research; (2) perform an assessment of output data from these devices to evaluate how comparable their outputs are. To this end, we chose one research-grade device (Empatica E4), one popular commercial-grade smart watch (Fitbit Sense), and one novelty ring device (Oura). Thus, the research questions are:

- RQ1. What are the characteristics of the native data from the different types of devices?
- RQ2. How well does the data output correlate between the devices?
- RQ3. How well does the device data output reflect self-reported symptoms and activities?
- RQ4. Which type of device has the necessary physical characteristics for practical use in PD research?

## Results

**Fig. 1:**
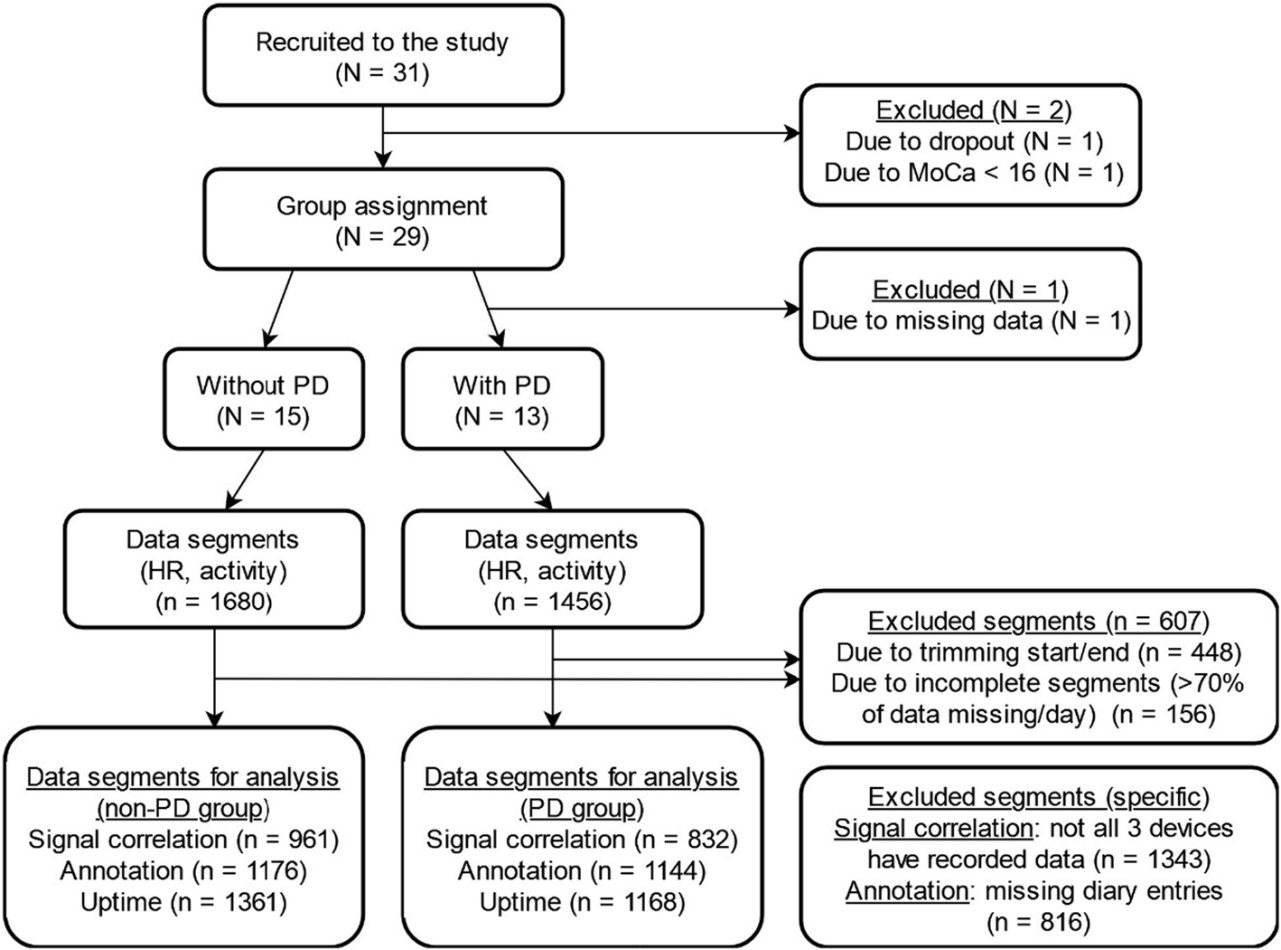
Flowchart of participants and data segments

A total of 31 participants were screened for participation in the study, and 28 were included in the PD group (*N* = 13) and in the non-PD group (*N* = 15). Figure 1 shows the flowchart of participants and the continuous monitoring data segments, resulting from the process of applying the inclusion/exclusion criteria and methodology, respectively.

The demographic information and the clinical assessment scores at baseline and end of study are presented in Table 1. The two groups were balanced for age, but not gender. The PD group had a Hoehn and Yahr stage of 1.92 ± 0.26, indicative of moderate disease severity. MDS-UPDRS III was slightly higher at baseline compared to the end of study due to a longer interval from last medication dosing. The PD group had a slightly lower MoCA score, which slightly improved in both groups at the last visit, as a result of a learning effect.

**Table 1:** Demographics and baseline clinical assessment scores

Characteristics and scores	PD group (*N* = 13)		Non-PD group (*N* = 15)	
	Baseline	End of study	Baseline	End of study
Age	70 (7.73)		72 (9)	
Gender: male (female)	10 (3)		4 (11)	
Hoehn and Yahr	1.92 (0.26)	1.84 (0.36)	–	–
MDS-UPDRS III	31.4 (13.9)	21.9 (6.8)	3.93 (4.1)	4.6 (4.1)
Medication time	173 (179.7)	116 (93.9)	–	–
MoCA	24.4 (2.95)	25.8 (3.8)	25.2 (1.83)	26 (2.5)
RBDSQ	5 (4.13)	4.4 (3.52)	2.92 (2.73)	3 (3.1)
Handedness: right (left)	12 (1)		14 (1)	

Values are given in mean and standard deviation in parenthesis, except for gender

*PD* Parkinson’s disease, *UPDRS* Unified Parkinson’s Disease Rating Scale, *MoCA* Montreal Cognitive Assessment scale, *RBSDQ* REM Sleep Behavior Disorder Screening Questionnaire; Medication time: minutes since last levodopa dose, *SD* standard deviation, – not collected

Table 2 presents the device and data characteristics, as well as the results of the implementation assessment. During this step, we determined that not all sensor data were comparable across all three devices. For instance, Fitbit Sense and Oura are equipped with 3-axis accelerometers, but the raw data are not saved and thus not available for download. Therefore, based on the types of sensors and the accessible signals from the devices, we selected the HR and activity (MET and MOV) as variables, because these two types are used to track behavioral trends over time. The MET and MOV quantify the level of physical activities, while the HR reflects the physiological response of the body to these activities. While HR is not usually found in the tracking of PD symptoms, we believe it provides valuable information on the overall health status of the participants [35]. The HR is provided by all three devices in bmp (beats per minute), while the activity is provided as MET scores for Fitbit Sense and Oura, and as 3-axis accelerations for Empatica E4 (either raw for annotation or as the aggregate MOV).

**Table 2:** Device and data characteristics, and implementation assessment results

Characteristics	Empatica E4	Fitbit sense	Oura
Processing level	Raw and aggregate	Aggregate	Aggregate
Sampling rate (for compared variables)	1 Hz for HR and 32 Hz for acceleration	Average sampling rate of 0.2 Hz for HR, but varies based on type of activity (rest/active), and 0.016 Hz for MET	0.0033 Hz for HR and 0.016 Hz for MET
Outcome availability for continuous monitoring	All	HR (rest are aggregates)	HR (rest are aggregates)
Raw data	HR, BVP, IBI, EDA, temperature, 3-axis acceleration	–	–
Aggregates (minutes)	–	HR, MET level, MET minutes, calories, steps	HR, HRV, MET level, MET minutes, hypnogram
Aggregate (daily)	–	–	Respiration, sleep stage duration, temperature, steps, calories, activity calculations
Filetype	CSV	CSV	CSV, JSON
Data ready for analysis	Need restructuring	Need restructuring	Need restructuring
Volume per 14 days	Approx. 750–1000 MB	Approx. 3 MB	Approx. 0.3 MB
Cleaning the device	Difficult	Easy	Easy
Device dependency	Operational without phone, tablet, or computer	Required to connect to phone or tablet for activation. Operational without phone afterwards	Required to connect to phone or tablet for activation. Not operational without phone afterwards due to lack of screen
Data retrieval	Bluetooth (only for 24-h of data) and USB cable via web platform	Bluetooth via web platform	Bluetooth via web platform
Mounting	Difficult wrist strap latching	Easy to latch	Easy to use, but different fingers had to be chosen due to fixed ring sizes
Data storage^a^	Cloud storage	Cloud storage	Cloud storage
Data transfer^a^	USB 2.0, Bluetooth	Bluetooth 5.0, Wi-Fi chip, and NFC chip	Bluetooth
Charging time	< 2 h	45 min	20–80 min
Battery life^a^	48 h	72–144 h	72–168 h
Charging type^a^	USB micro	Magnetic + USB 2.0	Magnetic + USB C-type
Data processing level	Raw signal data	Processed data	Processed data
Storage capacity^a^	Approx. 60 MB	4 GB	0.5 MB (V2), 16 MB (V3)
Water resistant^a^	No	Yes	Yes

*HR* heart rate, *MET* metabolic equivalent task, *BVP* blood volume pulse, *IBI* inter-beat interval, *EDA* electrodermal activity, *HRV* heart rate variability, *CSV* comma-separated values, *JSON* JavaScript object notation, *MB* megabyte, *GB* gigabyte, *USB* universal serial bus, *NFC* near-field communication

^a^Reported by manufacturer. The characteristics “cleaning the device” and “mounting” are based on the experience of the researchers when using the device throughout the study. “Activity calculations” is a term used to cover a range of activity states. Volume per 14 days is an approximate value based on the data we collected throughout the study period

**Fig. 2:**
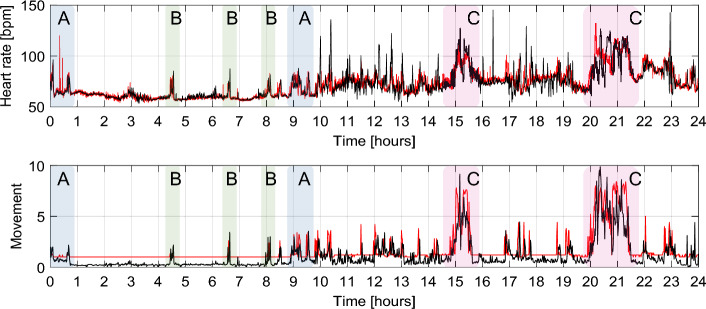
Visualization of four 24-h data segments from Empatica E4 and Fitbit Sense. Top: HR (in bpm). Bottom: movement as acceleration aggregation MOV (in m/s^2^) from Empatica E4 and scaled metabolic equivalent task (in METs) from Fitbit Sense. Annotations: A (light blue) for the sleep–wake cycle, B (green) for sleep disruptions, and C (pink) for activities during the day (walks)

**Fig. 3:**
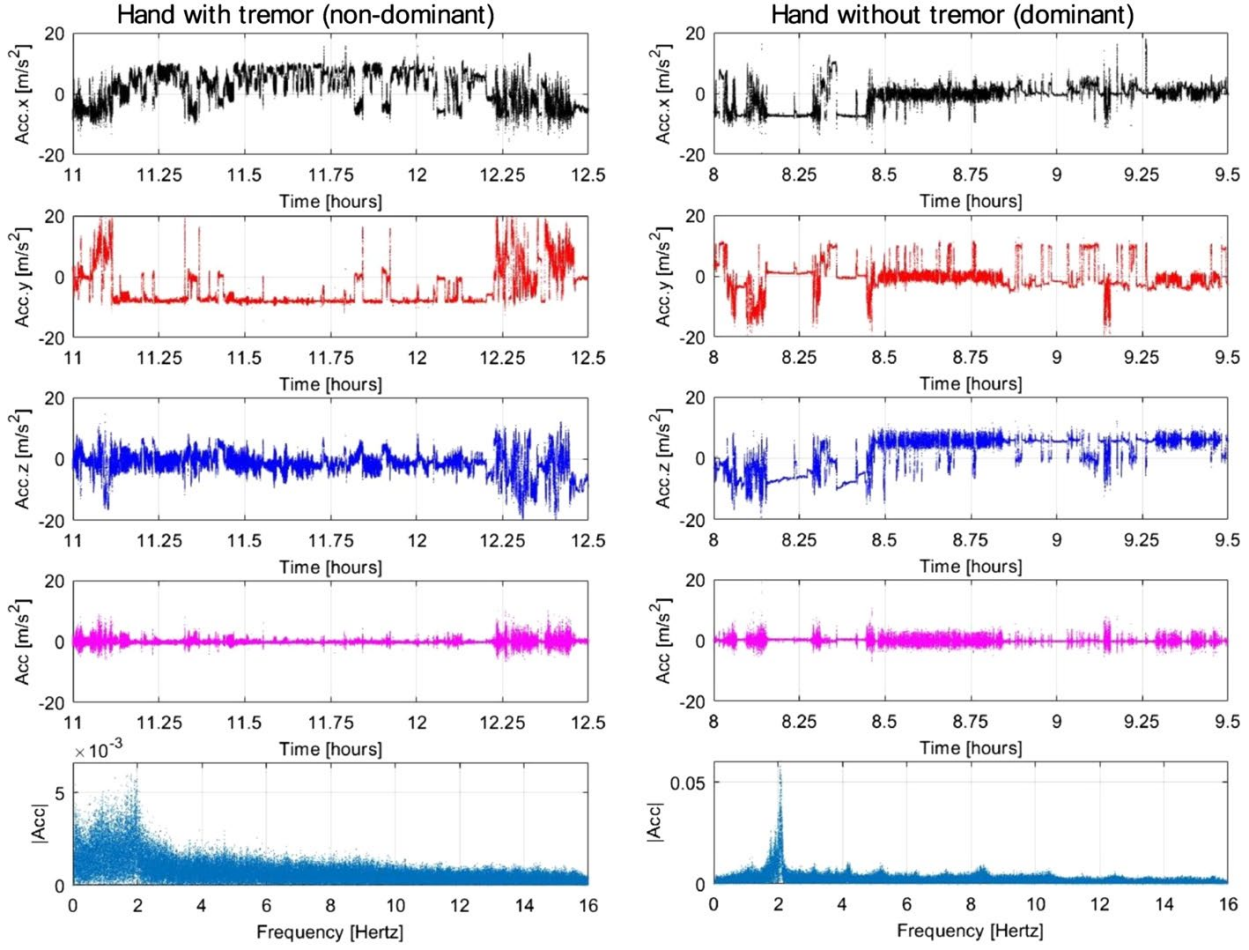
Visualization of Empatica E4 3-axis acceleration segments of 1.5 h each, from one participant with PD, for both the hand affected by tremor symptoms (left, non-dominant) and the hand not affected (right, dominant). Top to bottom: (1–3) acceleration on each axis, (4) Euclidean norm of (1–3) without the mean component, (5) Fast Fourier Transform of (4)

Figure 2 shows an example of data annotation and visualization for one participant and one day (midnight to midnight), in which areas are annotated after being identified as the beginning and end of the sleep cycle, sleep disturbances, and activities, which were then compared to the diary self-reports. Figure 3 illustrates two 1.5-h periods from one participant’s two hands: the non-dominant hand was reported as experiencing tremor, while the dominant hand was most active during working time (according to the diary). Thus, the overall daily behavior trends have a higher correspondence to the diary self-reports, while the tremor symptom was more difficult to discern visually by the rater.

The signal correlation analysis results are presented in Table 3. Results show that there is in general a weak correlation between the three devices. The HR outputs from Empatica E4 and Fitbit Sense show smaller differences in terms of NRMSE, while the movement-based MET/MOV show up to 20% difference. The Pearson coefficient valued at around 0.42–0.63 shows little similarity between the shapes of the signals, i.e., the variations in HR are not recorded simultaneously by the two devices, even though they are mounted on the body, monitoring the same cardiovascular system. While Pearson’s coefficient removes the mean, the cross-correlation coefficient includes the mean of the signals and is thus higher in value, confirming the results illustrated by the NRMSE: values are similar, but shape is not. An example is depicted in Fig. 2, HR section, during activities annotated C.

**Table 3:** Signal correlation analysis results: Pearson’s correlation coefficient, the normalized cross-correlation, and the normalized root mean of square error

Signal pairs	PD group			Non-PD group		
	Pearson’s Coeff	Cross-corr	NRMSE	Pearson’s Coeff	Cross-corr	NRMSE
HR	(*n* = 1,769,053)			(*n* = 2,316,481)		
Fitbit Sense vs. Empatica E4	0.54	0.97	0.09	0.59	0.97	0.09
MET/MOV	(*n* = 147,436)			(*n* = 179,644)		
Fitbit Sense vs. Empatica E4	0.50	0.73	0.12	0.42	0.68	0.20
Oura vs. Empatica E4	0.56	0.74	0.20	0.47	0.68	0.20
Fitbit Sense vs. Oura	0.61	0.80	0.13	0.63	0.81	0.16

All *p*-values = 0.000

*PD* Parkinson’s disease, *HR* heart rate, *MET* metabolic equivalent task, *MOV* Empatica E4 movement aggregate score, *NRMSE* normalized root mean of square error, *n* number of datapoints (per signal in pair) after resampling over 832 segments (days, PD group) and 961 segments (days, non-PD group)

The mean fit scores based on the manual annotation are presented in Table 4; the calculation includes the scores of 0 for device not recording. Results show a slightly better performance for HR in the Fitbit Sense data compared with Empatica E4, while the performance for movement was better for Empatica E4 compared to Fitbit Sense. The Oura data had the overall lowest fit scores. During annotation, we discovered that the MET and MOV signals were not detailed enough to allow for assessment of motor symptom reports, thus these were assessed only for the raw 3-axis acceleration from Empatica E4. Among these, tremor symptoms were the easiest to discern (although not with enough precision), while dyskinesia events were not possible to disambiguate from regular activities. Overall, the motor symptoms (tremor, bradykinesia, rigidity and dyskinesia) were the most challenging to evaluate visually by the human rater, while sleep disturbances were ostensibly more obvious.

**Table 4:** Fit scores means and standard deviations for the three devices and the two groups

Device fit scores	PD group (*n* = 1475)			Non-PD group (*n* = 1294)	
	HR	MET/MOV	Acceleration^a^ (raw)	HR	MET/MOV
Empatica E4: mean (SD)	3.91 (1.42)	4.10 (1.33)	2.26 (1.80)	3.71 (1.45)	3.72 (1.40)
Fitbit Sense: mean (SD)	4.05 (1.41)	3.85 (1.55)	–	3.92 (1.25)	3.87 (1.30)
Oura: mean (SD)	1.20 (1.89)	2.50 (2.03)	–	2.27 (2.05)	2.39 (2.13)

*PD* Parkinson’s disease, *HR* heart rate, *MET* metabolic equivalent task, *MOV* Empatica E4 movement aggregate score, *SD* standard deviation, *n* number of annotations (total per device)

^a^582 annotations

The differences between devices for the PD and non-PD groups for both HR and MET/MOV are presented in Table 5, as percentage of zero differences (i.e., amount of score similarity). Results show significant differences between Empatica E4 and Oura, and between Fitbit Sense and Oura, for both HR and MET/MOV. Additionally, a significant difference is shown for HR between Empatica E4 and Fitbit Sense in the PD group.

**Table 5:** Percentage of zero differences between devices for HR and MET/MOV

Differences between devices: zeroes [%]	PD group (*n* = 1144)		Non-PD group (*n* = 1361)	
	HR	MET/MOV	HR	MET/MOV
Empatica E4–Fitbit Sense	53.8 (*p* = 0.0092)	53.1 (*p* = 0.4638)	69.3 (*p* = 0.0725)	62.5 (*p* = 0.1048)
Empatica E4–Oura	11.1 (*p* = 0.0000)	18.8 (*p* = 0.0000)	19.7 (*p* = 0.0000)	25.8 (*p* = 0.0000)
Fitbit Sense–Oura	11.8 (*p* = 0.0000)	24.4 (*p* = 0.0000)	17.6 (*p* = 0.0000)	32.6 (*p* = 0.0000)

*PD* Parkinson’s disease, *HR* heart rate, *MET* metabolic equivalent task, *MOV* Empatica E4 movement aggregate score, *n* number of segments (days, total), which is divided for the sign test in segments (days) per device, per output signal (HR, MET, acceleration) *n* = 143 (PD group) and *n* = 147 (non-PD group)

The total annotatable data (Table 6) varies across devices and data streams, showing that Empatica E4 had the highest amount of annotatable data for the non-PD group, closely followed by Fitbit Sense. Results were reversed for the PD group. Oura had the least annotatable data.

**Table 6:** Percentage of data available for manual annotation and uptime calculation

Device	PD group		Non-PD group	
	HR	MET/MOV	HR	MET/MOV
Annotatable data [%]	(*n* = 1144)		(*n* = 1361)	
Empatica E4	93.83	95.00	91.98	93.50
Fitbit Sense	96.91	96.56	92.90	93.64
Oura	59.88	63.44	32.19	66.24
Inter-day uptime [%]	(*n* = 1168)		(*n* = 1361)	
Empatica E4	98.08	100.00	93.18	100.00
Fitbit Sense	94.87	94.87	94.89	84.97
Oura	80.13	80.77	89.20	92.74
Intra-day uptime [%]	(*n* = 1168)		(*n* = 1361)	
Empatica E4	95.53	99.79	84.67	100.00
Fitbit Sense	99.97	94.52	87.58	94.42
Oura	88.48	78.74	76.78	90.69

*PD* Parkinson’s disease, *HR* heart rate, *MET* metabolic equivalent task, *MOV* Empatica E4 movement aggregate score, *n* number of segments (total, days)

The mean SUS scores and standard deviations (*N* = 4 researchers) are: 37.5 (0.86) for Empatica E4 indicating a poor usability, 70 (1.1) for Fitbit Sense indicating good usability, and 60.62 (1.28) for Oura indicating an “okay” usability [36]. Empatica E4 is described by the researchers collecting data as challenging to use when latching, uploading data, cleaning and charging. The device has many small crevices where dry sweat and skin would collect, and with the device not being waterproof, it made cleaning difficult. Participants in the first collection wave who were asked to charge the device themselves were unable to do so reliably, due to difficulties in connecting the charger, which triggered a more time-consuming re-design of the data collection procedure. While Fitbit Sense has an easy strap to operate both for researchers and participants, Oura’s ring rigidity can complicate trial designs when multiple participants require the same ring size at the same time. However, both Fitbit Sense and Oura have an intuitive design for both charging and mounting. All three devices have had challenges when uploading data, ranging between manual procedure, not synchronizing, requiring connection to power source, debris on connectors, etc.

### Measurement errors and challenges

(1) Empatica E4: During the annotation process, the Empatica E4 HR signal showed measurement errors when devices were reported as removed from the wrist (e.g., for showering), displaying values of over 200 bpm which are implausible for these participant groups. Upon this discovery, we chose to continue following our data analysis methodology (as described in "Methods") and keep these implausible values, because the aim of this study is to assess the native (out-of-the-box) data of these devices, on a level that does not require further software development. Moreover, several participants ended sessions when intending to mark events, resulting in gaps in the data. The data upload would sometimes disconnect when multiple devices were connected at once. Uploading would sometimes fail and customer support had to be contacted. During use, some participants noted that the strap gave them a rash. Others complained about difficulty latching the strap, and one participant reported light from sensors waking them up. (2) Oura: The ring data showed discrepancies between non-wear values in MET scores vs. measured HR during sleep.

## Discussion

In this study, we investigated the utility of three wearables—Empatica E4, Fitbit Sense and Oura—for PD research, focusing on their data output. Our analysis revealed significant variation in the data characteristics and a low correlation between devices. Empatica E4 and Fitbit Sense demonstrated better alignment with self-reported activities compared to Oura. Through this assessment, we identified key features that wearables for PD research should have, which were collectively represented across the three devices. We concluded that while no single device fully meets all research and development requirements, each has valuable technical or practical strengths, which offer potential for improvement.

The devices varied in terms of access to data output, sampling rate and level of device-native data processing, ease of use and retrieval of data, and design. Oura and Fitbit Sense were durable, waterproof, and easy to mount, charge, clean and use. Empatica E4 was more cumbersome to use, had a bulkier design, challenging to mount, charge, clean and use. Design features such as being lightweight, comfortable, easy to use, unobtrusive are highlighted as important for PD research [15, 27]. Noteworthy, the Empatica E4’s successor, the Embrace Plus has a different design [37]. Depending on outcome measures, the three wearables can provide different resolutions of measurement, in which the research-grade data are useful for investigating motor symptoms, while the commercial-grade aggregates can track overall behavioral trends of sleep and daily activities. None of the devices provided native calculations for PD symptoms specifically, which, on the one hand opens up possibilities for further development of transparent assessment tools, but on the other hand requires interdisciplinary resources and dedicated research efforts.

The wide variation in battery life can affect how a study is conducted when including older adults, especially with neurological conditions such as Parkinson’s disease. This is due in part to reduced digital literacy and function, i.e., some participants might have difficulties in charging the devices by themselves [38, 39]. In our study, we have encountered this very problem, which then informed the design of the data collection schedule. For very large studies that are geographically distributed, this may pose an obstacle. At the same time, even for devices with longer battery life, the collection of high-frequency data might be affected by the limitations of the available storage. With important differences in the proprietary platforms for data download, we believe it is unrealistic to expect that all study participants might manage the device during long periods of data collection without support.

The effect of the device placement on HR and activity (MET and MOV) has been considered during and is partly reflected in the signal correlation analysis. For HR, we performed the comparison between Empatica E4 and Fitbit Sense, the former having been shown to provide measurements independent of side and handedness [40]. To account for inter-hand variations and lateralization of symptoms, we calculated both Pearson’s coefficient and NRMSE, which together describe similarity in shape (longitudinal match) and values (vertical match), as shown in Table 3. Thus, the HR values are close (NRMSE = 0.09), while their shape differs (Pearson’s coefficient = 0.54–0.59), confirmed by the cross-correlation coefficient (0.97).

For activity, the participants’ handedness and/or lateralization may have impacted the comparison. Earlier research found differences between the dominant vs. the non-dominant hand on some devices [41]. Similarly, step estimation accuracy has been found to depend on PD symptoms and medication status [42]. As with the HR analysis, we use Pearson’s coefficient, the cross-correlation coefficient, and NRMSE to account for these variations. Results show low correlation between Empatica E4 and Oura, worn on the same hand, for which we would expect marginal differences (due to finger vs. wrist mounting), regardless of lateralization or handedness. This indicates that native calculations of commercial devices are not necessarily interchangeable (e.g., MET vs. MOV) and could affect activity tracking over time. Interestingly, the Fitbit Sense vs. Oura pair showed the highest correlation out of all pairs, despite being worn on opposite sides, likely due to the nature of MET scores; these are proprietary to both manufacturers, and the underlying calculation methods cannot be compared. We surmise that future studies should investigate the effect of lateralization, handedness and participant preference on various permutations before choosing a device configuration.

The low correlation between HR signals for the three devices is concerning. While Empatica E4 produced raw HR data, it also assigned erroneous values (e.g., 200 bpm) when the devices were not worn. Fitbit Sense HR appeared more accurate, but the data did not reflect HR at constant measurement frequency throughout the day; instead, the device returned an aggregate at a priori undisclosed time resolutions, which ultimately affected how this data could be analyzed. Oura V2 only measured HR during rest, but when the continuous HR measurement service was introduced in version V3, it came with additional subscription costs. Differences in HR measures have been reported among commercial-grade devices [43], even between devices from the same manufacturer [44]. Comparatively, the Empatica E4 sensor has been constantly obscured by skin and sweat residue, whereas Fitbit Sense and Oura had a smooth glass coating over the sensing elements.

For activities, we found large discrepancies between Oura and the other two devices, which could be due to the ring being worn on the finger and the watches on the wrist. Even so, tracking activity as a MET score is insufficient for research, as MET is an estimate of energy expenditure [45] and not movement directly, whereas the MOV score we calculated is an aggregate of the raw acceleration signal [46]. MET is prone to error [47] due to individual differences in resting metabolism, body-mass index and age [48], and therefore the MOV metric has mirrored bouts of activity reported in the diary more closely.

Interestingly, the annotatable data from Oura (30% for HR and 65% for MET) provides insight into the average hours of sleep and wakefulness among older adults [49]. However, the limitation of only measuring HR during sleep and MET while awake, makes the Oura ring less reliable for RBD research. Individuals with PD typically experience altered sleep [50] with fewer total sleep hours and fewer consecutive hours of sleep [51], resulting in more awakenings, and, consequently, more interruptions in HR recording from Oura. These interruptions are not due to the device detecting sleep or wake states but are caused by movement of the ring. On the positive side, the ring’s shape may make it more suitable for sleep-related research, as it is more comfortable to wear at night compared to wristbands.

The three devices are not interchangeable and future PD applications should carefully consider factors such as hardware and software reliability, data quality, whether validation studies have been performed, transparency from manufacturers and, the commercial practices of these companies to avoid hidden costs. Moreover, access to raw data is crucial for research, where new indicators or scores need to be designed and validated, which commercial-grade devices currently do not provide.

Trends such as movement during sleep time, awakenings, sedentary behavior, general activity level, walks and exercise can be tracked using aggregated data available from commercial devices [52, 53] and may have utility for clinical practice [32]. Although Fitbit Sense only provided aggregate data, it does allow for extracting raw accelerometer data using custom software. This acceleration data have been compared to research-grade devices and produced comparable output results [54]. Raw accelerometer data from wearables over long periods have shown that severity of disease is associated with less time walking [55], and machine learning models trained on accelerometry data have been found to outperform models trained on measurement modalities such as genetics, lifestyle, blood chemistry in predicting prodromal PD up to 7 years pre-diagnosis [51]. The utility of acceleration data in general is promising. The potential of acceleration data is promising, but a key challenge with Fitbit Sense is that researchers must develop custom software to access and record the raw data, which can be both costly and delay data collection. Lastly, access to raw data from all sensors embedded in a device and transparency of how the device operates is crucial for accurate and reproducible science, especially science involving the potential clinical outcomes of patients in the future. When it comes to symptom tracking from raw data, visualization is not enough, and building algorithms to quantify these features is a more adequate approach to symptom tracking.

Although designing new devices tailored for research is possible [56], utilizing already existing ones is preferable, not only to save costs, but also for environmental reasons. However, beyond issues of data access and transparency, these already existing devices suffer from planned obsolescence [57], and changing the device mid-collection may cause validity issues in clinical trials. Ideally, new digital measures would be designed together with the hardware (e.g., smartwatch), but this requires considerable interdisciplinary collaboration and lengthy medical approval processes.

### Limitations

The addition of a control group (non-PD) allowed us to explore differences between groups in terms of benefits, challenges, and output for the devices. During the study period, Oura V2 was phased out for the newer version V3. The fit score used to rate the congruency between self-reported diary and symptoms visualized through device data is a subjective assessment. The annotation process had only one rater, to avoid inter-rater bias, which can ultimately reflect in an offset for the fit scores.

This study is centered around the cross-evaluation of data from the three wearable devices. Due to the fact that most outcome measures were device-specific and not provided by all three, the comparative assessment was limited to two signals (HR and MET/MOV). Therefore, we did not assess the additional functionality or potential for multi-modal sensing that might be possible by considering, e.g., EDA, HRV, number of steps, or temperature.

The diary for the PD group was much more open, allowing participants to explain what symptoms they had and how they felt it impacted them, but this also made it more prone to difference between participants, whereas the diary for the non-PD participants was more structured and shorter to administer. Neither of these diaries were validated instruments. Visiting participants every second day was laborious, but was perceived as a nice experience for both the researchers and, more importantly, the participants, giving them a chance to at length talk about their condition and how it affects their lives. Many participants expressed great appreciation for the informal conversations and visits from the researchers.

## Conclusions

In this study, we evaluated three commercial and research-grade wearable devices with the aim of investigating their utility in PD research and performing an assessment on their output data. We ultimately identified necessary characteristics for PD research, such as ease of handling, cleaning, and data retrieval, access to raw data, score calculation transparency, long battery life, sufficient storage, higher sampling frequencies. None of the three devices were deemed optimal for PD research, but they all present reasonable qualities, meaning that future iterations could provide suitable specifications. But planned obsolescence is an issue across the board, which can cause validity problems in PD trials, and so we conclude that more restrictive policies for wearable development are required, especially for use in healthcare.

## Methods

### Participants and setting

This work includes data from all eligible participants in the ActiveAgeing study, formed of the DIGI.PARK and Helgetun branches. The participants in the DIGI.PARK branch are individuals with PD recruited from the STRAT-PARK cohort (*N* = 15) [58], while the control group comprised older adults without PD from the Helgetun branch (*N* = 16) [59]. The inclusion and exclusion of participants is: (a) diagnosis of PD (must have for the PD group and must not have for the non-PD group); and (b) recruitment location for the Helgetun branch. A detailed description of the ActiveAgeing study has been published [60].

### Outcome measures

#### Clinical assessment scales

The MDS-UPDRS is a clinical measurement tool for motor symptoms in PD consisting of four parts, out of which we use part III, an 18-item observer-rated motor examination on symptom types and severity (range 0–4) [61]. The Montreal Cognitive Assessment scale (MoCA) is a validated cognitive screening tool used to detect mild cognitive impairment [62]. The REM Sleep Behavior Disorder Screening Questionnaire (RBDSQ) is a tool used for screening rapid eye movement (REM) sleep behavior disorder, validated in both people with and without PD [63, 64], consisting of 13 questions and Yes/No responses. The validated Norwegian language versions of MoCA [65] and RBDSQ [66] are applied in this study. The Hoehn and Yahr Scale [61] is used to describe the functional disability associated with PD, consisting of five stages from minimal to severe disability.

#### Self-reported measures

A diary was constructed to log the occurrences of various activities, symptoms (tremor, dyskinesia, bradykinesia, rigidity, stiffness of gait, balance), sleep schedule, sleep disturbances, medication times, symptom lateralization, and handedness. For the PD group, the diary is divided into 30-min intervals, with the severity of motor symptoms reported on a scale of mild to severe. Fluctuations of ON/OFF states caused by variations in dopamine levels are also logged. For the non-PD group, the diary is divided into 24-h intervals and is structured as a questionnaire (Additional file 1).

#### Digital measures

Empatica E4 is a research-grade wrist-worn multi-sensor device with a single button used for starting device and recording, marking events, and ending recording [67]. The device measures heart rate (HR), inter-beat interval (IBI), blood volume pulse (BVP), movement, electrodermal activity (EDA) and body temperature (Table 7). Empatica E4 has mixed results for the validity and reliability of both HR and EDA [40, 68–70].

**Table 7:** Description of devices and their sensors, outputs, and sampling rates

Sensor type	Device					
	Empatica E4		Oura ring		Fitbit Sense	
	Sensor, frequency	Output	Sensor, frequency	Output	Sensor, frequency	Output
Accelerometer	✓^a^, 32 Hz^a^	Raw data^a^	✓, ^b^	Sleep^a^	✓, ^a^	–^b^
Gyroscope	–	–	–	–	✓, ^a^	–^b^
Altimeter	–	–	–	–	✓, ^a^	–^b^
EDA sensor	✓*, 4 Hz^a^	Raw data^a^	–	–	✓, ^a^	–^b^
PPG/BVP	✓*, 64 Hz^a^	Raw IBI/HRV, HR^a^	✓, 50 Hz^c^	Sleep^a^, HR^d^, HRV^a^	✓, ^a^	Calories, MET^a^
Light sensor	–	–	–	–	✓, ^a^	–^b^
Temperature	✓*, 4 Hz^a^	Raw data^a^	✓	Sleep^a^	–	–

*Hz* hertz, *MET* metabolic equivalent task, *IBI* inter-beat interval, *HR* heart rate, *HRV* heart rate variability, *EDA* electrodermal activity, *PPG* photoplethysmography, *BVP* blood volume pulse

^a^Reported by the manufacturer

^b^Not reported by the manufacturer

^c^Only for version V3

^d^Only during sleep

Oura is a consumer-grade multi-sensor smart ring, which provides several aggregated scores, including hypnogram (sleep), heart rate variability (HRV), HR and metabolic equivalent task (MET) (Table 7). The ring has shown promising results for measuring MET [71], HR during sleep [72], and mixed results for measuring sleep stages when compared to polysomnography [73, 74]. Both version V2 and V3 are used, due to V2 obsolescence during the study period.

Fitbit Sense is a consumer-grade multi-sensor smartwatch that outputs several aggregated scores, including HR, energy expenditure (calories), MET and number of steps (Table 7). Fitbit wearables have been found to be the most utilized smartwatches for research in 2018 [75]. Fitbit Sense specifically has shown good agreement with validated devices for heart rate monitoring, but poor performance for monitoring energy expenditure [43].

Fitbit Sense and Oura ring were connected to a Samsung Galaxy Tab A7 running Android OS.

The selection of these three devices was informed by several factors: people with PD have shown high acceptability and compliance in studies using wrist-worn smartwatches [76, 77]; minimization of the number of sensors and their ease-of-access locations, as suggested by the roadmap for digital measures in PD [15]; the pervasiveness and availability of wrist-worn devices. As more people start wearing smartwatches [78], they will become a staple in daily life, and as such, it is of high interest to investigate whether they can fulfill the additional function of symptom monitoring over time, despite their obvious limitations: unilateral motion assessment as the smartwatch is worn on one arm, noisy measurements due to daily life activities, or the challenges of estimating postural parameters from the movement of the wrist.

### Data collection procedure

**Fig. 4:**
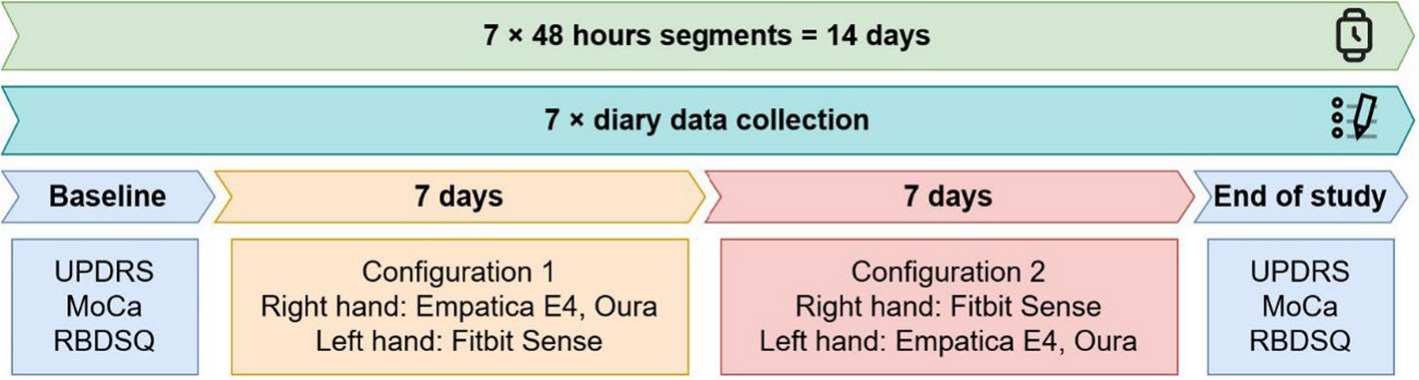
Data collection timeline. *UPDRS* Unified Parkinson’s Disease Rating Scale, *MoCa* Montreal Cognitive Assessment scale, *RBSDQ* REM Sleep Behavior Disorder Screening Questionnaire

The duration of data collection for each participant is 14 days. Researchers visited the participants in their homes every 48 h to perform data collection and device maintenance. The non-PD group was structured in consecutive waves of 5 participants, between 12.2021 and 03.2022. The PD group data collection took place asynchronously for the participants, between 03.2022 and 12.2022. The data collection timeline is described in Fig. 4:

- Clinical assessment scales were applied at baseline and at the end of study (14 days).
- Diaries (self-reported measures) were collected every second day. Participants were advised to keep track of their days on their own, by writing down events, and then were asked to summarize their days during the data collection visit.
- *Wearable devices*. Participants wore the three devices simultaneously. Participants in the non-PD group wore the Empatica E4 and Oura ring on the right hand and Fitbit Sense on the left hand. Participants in the PD group switched hands for the second week (Fig. 4).

The mounting configuration of the three devices is informed by their type and the usual placement choice of regular wrist watches or similar commercial smartwatches. This is because the Empatica E4 is embedded in a non-transparent box, whereas the Fitbit Sense has a screen with a watch face; thus, we are able to compare Empatica E4 and Fitbit Sense as a research-vs.-commercial-grade pair. Oura is placed on the same side as Empatica E4 to allow for a finger vs. wrist comparison. To capture the lateralization of PD-related symptoms, this configuration is applied to both sides of the body in the PD group. The device placement, symptom lateralization and handedness are recorded in the diary.

### Analysis

**Fig. 5:**
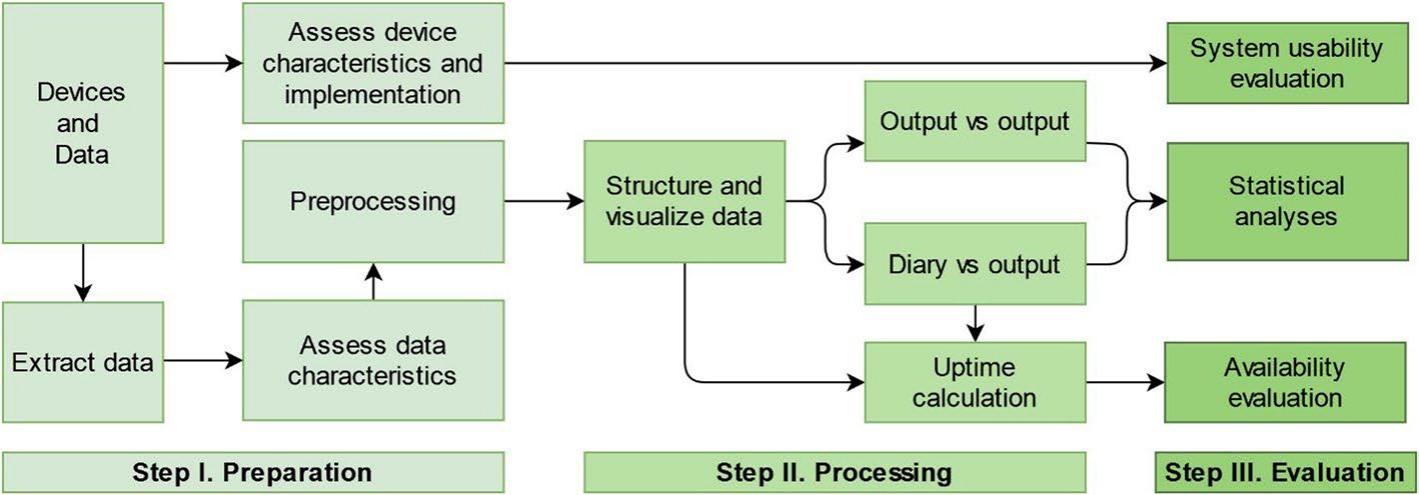
Analysis steps for the cross-evaluation of the three devices

The analysis for the cross-evaluation of the three devices is structured over three steps, as shown in Fig. 5: I. preparation, II. processing, and III. evaluation.

Step I. *Preparation*. This analysis stage covers the assessment of device characteristics and the implementation process, as well as the pipeline for data extraction and preprocessing.

To assess device characteristics, we inspect the design features such as shape, size, mounting type, buttons, and screen. The implementation assessment covers the ease of use in terms of charging, connecting to tablet or computer, retrieving data, cleaning the device, and mounting on intended body part. Moreover, we assessed the battery life of devices and memory capacity during the first data collection wave of the non-PD group (*N* = 5).

We extract data through the proprietary dashboards that each device had as a default. We then assess the characteristics of the data in terms of resolution (sampling rates), processing level (on a scale from raw to aggregated), volume, type of sensor outcome measures (e.g., acceleration in m/s^2^, sleep stages, etc.), filetype and format. Based on these characteristics, we choose the outputs which are comparable across devices to utilize in the next two steps.

During preprocessing we: standardize the timestamps to Unix time, segment the data into 24-h segments (00:00:00–23:59:59), and convert filetype to MATLAB-compatible types.

Step II. *Processing*. This stage covers data structuring and visualization, cross-correlation between outputs, comparison with diary and the uptime calculation.

For data structuring, we inspect each segment and exclude the incomplete days (due to pause in data collection and partial days from segmenting), if more than 70% of the data from at least one device is missing. We visualize the data by plotting each segment across the three devices, per outcome measure, as follows: HR is plotted for all three devices, 3-axis acceleration for Empatica E4, and MET (Fitbit Sense and Oura) vs. movement (MOV) (Empatica E4). MOV is an aggregated movement score calculated based on the 3-axis acceleration from Empatica E4, calculated with a sampling time of 1 min (to match the sampling time of MET from Fitbit Sense and Oura):

1 $${\text{MOV}}_{i}=\frac{1}{m}\sum_{k=1}^{m}\text{max}\left(\left|{x}_{k}-{x}_{k-1}\right|,\left|{y}_{k}-{y}_{k-1}\right|,\left|{z}_{k}-{z}_{k-1}\right|\right) ,$$

where *x*, *y* and *z* are accelerations on the three axes, *k* is current datapoint (sampling rate of the raw acceleration), and *m* is the number of concurrent samples per minute (i.e., for one axis).

To compare device outcome measures (outputs) against each other, we perform a signal cross-correlation analysis for which we calculate Pearson’s correlation coefficient [79], the normalized cross-correlation [80], and the normalized root mean of square error (NRMSE) [81]. We perform the cross-correlation calculation per participant category (PD and non-PD groups) between (1) HR from Fitbit Sense and Empatica E4 (Oura is excluded due to device only recording during nighttime); (2) MET (Fitbit Sense and Oura) and MOV (Empatica E4). In signal processing, Pearson’s coefficient and the normalized cross-correlation are used to indicate the longitudinal match of two signals and is defined over [−1; 1], where 1 is best match, 0 is no match, and −1 is for mirrored signals. The NRMSE indicates the vertical match defined over [0; 1], where 0 is best match and 1 is worst. Because the three devices record data at different sampling rates, the signal correlation analysis requires resampling, for which we use the Fitbit Sense sampling rate as a reference. We resample the Empatica E4 HR output at 1 s, via timestamp alignment and omitting the non-overlapping values. The MET and MOV scores are sampled at 1 min, either by design (provided by the device) or calculation (for MOV). MET measures are normalized on a [0–10] scale to enable comparisons across devices and with the MOV aggregate. To calculate the cross-correlation related measures, we handle missing values by removing datapoint pairs when at least one of the datapoints is missing (due to, e.g., device not recording). Thus, we omit data gaps of different lengths during the 24-h period.

To compare diary data with output from devices, we use a manual annotation procedure, in which a rater (H.R.) judges whether a particular activity or symptom in the diary data corresponds to an expected change in the visualized sensor data. We perform manual annotation of the HR and MET for all devices, and the 3-axis acceleration signals from Empatica E4, assigning *fit scores* ranging between 0 and 5. The annotation procedure excludes the segments for which diary data are missing (annotation not possible). The fit scores represent how well the plotted signal fits the reported activity/behavior or symptom at that time. Scores 1–5 are assigned for low to high congruency. A score of 0 represents a missing recording for that specific diary entry. For instance, if a participant reports exercising between hours 13:00 and 14:00, and the HR and MET/MOV signals increase correspondingly during this time window, this yields a fit score of 5, representing high congruency. If the heart rate and activity are low during the time window, this yields a fit score of 1, representing low congruency. For PD motor symptoms and sleep disturbances, the fit scores are assigned based on their manifestation. For instance, tremor is expected to show as rapid fluctuation in the movement signals, while sleep disturbances appear during nighttime in HR and movement as sudden changes surrounded by “flat” zones. If in the diary a tremor episode is reported, the rater would then search for a higher density of movement during that time period compared to adjacent ones and score accordingly. In contrast, we expect that bradykinesia and rigidity would appear as less dense, i.e., characterized by slower and rarer movement of the wrist. Finally, dyskinesia is the most challenging to discern, as it is expected to show as sudden and repetitive spikes in movement, and so the rater assessed these movements comparatively to the rest of the day. Stiffness of gait and balance are not expected to be visually evident due to the smartwatch being worn on the wrist.

We calculate the amount of annotatable data as the percentage of 0 value fit scores to non-zeros (1–5). We calculate the uptime as two measures:

- Inter-day uptime is the percentage of days where at least one device is recording from the total number of days. Pauses in data collection (all three devices not recording, over 70% of missing data per segment, partial days due to trimming) are excluded from calculation. Inter-day uptime allows us to assess the reliability of the device recording over many days.
- Intra-day uptime is the percentage of total non-zero data points out of all expected data points (*N*_*E*_), per device. For Empatica E4, we estimate *N*_*E*_ based on the fixed sampling rate reported by the manufacturer (Table 7). For Fitbit Sense and Oura, we estimate *N*_*E*_ based on each participant’s average sampling rate. For Fitbit Sense and Empatica E4 we expect the recordings to be continuous 24-h, and for Oura we expect HR only during sleep and MET during waking life. The intra-day uptime allows us to evaluate the reliability of a device consistently and continuously recording data throughout the day, and to determine if there are sudden disruptions in recording.

Step III. *Evaluation*. This stage covers the evaluation of usability and availability, as well as the rest of the statistical analyses. The acceptability and compliance of people with PD in wearable-driven studies has been shown before as high [76, 77], and thus we focus on investigating the usability of the devices from the perspective of the researcher. Therefore, evaluate the devices with the system usability scale (SUS), which is an instrument validated for measuring the usability of a system [82]. SUS yields a score from 0 to 100, where high score means that a system has a high usability, and a low score indicates a low usability, with scores > 85 representing excellent [36]. Four researchers (HR, BM, MP, EF) completed three SUS questionnaires, one for each device, based on their interaction with the device-platform system. The results are aggregated by item-wise averaging. We use the sign test [83] to compare the day-averaged fit scores for HR and activity (MET, acceleration) outcomes between devices. Summary reports (mean, standard deviation) are used to describe the mean fit scores and signal correlation measures (Pearson’s coefficient and NRMSE) for the two groups. To estimate device availability and recording reliability, we evaluate the annotatable data percentages and the uptime.

#### Software and tools

Scripts developed in-house in Python (Jupyterlab v. 3.0.14) are used to retrieve data from Oura and Fitbit Sense, and to segment data from all devices. Data formatting, visualization, preprocessing, resampling, and signal correlation analysis are performed using MATLAB v. 2019a. The other statistical analyses are performed using StataSE v.18.

## Supplementary Information

Supplementary Material 1.

## Data Availability

The datasets used in the current study are protected under privacy regulations and can be made available in deidentified form by reasonable request to the corresponding author.
