# Supplementary material for: Cross-evaluation of wearable data for use in Parkinson’s disease research: a free-living observational study on Empatica E4, Fitbit Sense, and Oura
Source: Biomed Eng Online. 2025 Feb 21;24:22. doi: 10.1186/s12938-025-01353-0 (PMC11846298; doi:10.1186/s12938-025-01353-0)
Supplement: Supplementary file 1 — Supplementary Material 1. [file 12938_2025_1353_MOESM1_ESM.docx]

**Additional file 1: Diary structure**

**Part 1. Baseline questionnaire**

**What symptoms do you typically experience?**

1. ……..
2. ……..
3. ……..

**1. When and how often do they occur? ……**

Time of day:

Frequency:

Duration:

**2. When and how often do they occur? ……**

Time of day:

Frequency:

Duration:

**3. When and how often do they occur? ……**

Time of day:

Frequency:

Duration:

**When do you normally take your medication, and how long does it normally take for the medication to take effect?**

Time of day:

Onset of action:

**Do you experience OFF periods, and do they occur at specific times during the day?**

Answer:

**Do you experience ON periods, and do they occur at specific times during the day?**

Answer:

**What side of your body (Right or Left) does the disease effect the most?**

Answer:

**End of study question: Have there been any challenges with using the technology?**

Answer:

**Part 2. Template for the diary of participants with PD**

**Part 3. Diary for control participants (Helgetun branch)**

| Monitoring questions, Helgetun data collection 1 | | | | | |
| --- | --- | --- | --- | --- | --- |
| **1.** | **How physical active have you been?** | | | |  |
|  | Very little | Little | Average | Quite | Very active |
|  | 1 | 2 | 3 | 4 | 5 |
| **2.** | **What activities have you been doing?** | | | |  |
|  | (type of activity, and approx. time and length) | | | |  |
| **3.** | **Has your activity deviated for some reason?** | | | |  |
|  | No | Yes | In that case, how: (reason) | | |
|  | 0 | 1 |  |  |  |
| **4.** | **How well have you been sleeping?** | | |  |  |
|  |  |  |  |  |  |
|  | Very poorly | Poor | Average | Good | Very good |
|  | 1 | 2 | 3 | 4 | 5 |
| **5.** | **Have you felt rested?** | |  |  |  |
|  | No | Quite | Yes |  |  |
|  | 0 | 1 | 2 |  |  |
| **6.** | **How many times have you been waking up?** | | | |  |
|  | (number) |  |  |  |  |
| **7.** | **Have you gone to bed and gotten up at normal times?** | | | | |
|  | No | Quite | Yes |  |  |
|  | 0 | 1 | 2 |  |  |
| **8.** | **How stressed have you been feeling?** | | | |  |
|  | Not stressed | Little | Some | Very |  |
|  | 0 | 1 | 2 | 3 |  |
| **9.** | **Have you had any challenges with the devices?** | | | | |
|  | No | Yes | Explain: |  |  |
|  | 0 | 1 | (challenges) | |  |
| **10.** | **Have you removed the devices at some point?** | | | |  |
|  | No | Yes | When: |  |  |
|  | 0 | 1 | (time) |  |  |

**Part 4. Template for the diary of participants without PD**

| **Participant ID** | |  |  |
| --- | --- | --- | --- |
| Date: |  |  |  |
|  |  | Week one |  |
|  | Days: | 1 | 2 |
|  |  |  |  |
| 1. | Activity level |  |  |
| 2. | Activities |  |  |
| 3. | Activity deviation |  |  |
| 4. | Sleep quality |  |  |
| 5. | Rested |  |  |
| 6. | Awakenings |  |  |
| 7. | Normal sleep routine |  |  |
| 8. | Stress level |  |  |
| 9. | Challenges devices |  |  |
| 10. | Removal of devices |  |  |
